# Serological Survey of Hantavirus in Inhabitants from Tropical and Subtropical Areas of Brazil

**DOI:** 10.1155/2016/8628949

**Published:** 2016-03-10

**Authors:** Felipe Alves Morais, Alexandre Pereira, Aparecida Santo Pietro Pereira, Marcos Lazaro Moreli, Luís Marcelo Aranha Camargo, Marcello Schiavo Nardi, Cristina Farah Tófoli, Jansen Araujo, Lilia Mara Dutra, Tatiana Lopes Ometto, Renata Hurtado, Fábio Carmona de Jesus Maués, Tiene Zingano Hinke, Sati Jaber Mahmud, Monica Correia Lima, Luiz Tadeu Moraes Figueiredo, Edison Luiz Durigon

**Affiliations:** ^1^Laboratório de Virologia Clínica e Molecular, Departamento de Microbiologia, Instituto de Ciências Biomédicas, Universidade de São Paulo, 05508-900 São Paulo, SP, Brazil; ^2^Laboratório de Sinalização Molecular, Departamento de Bioquímica, Instituto de Química, Universidade de São Paulo, 05508-000 São Paulo, SP, Brazil; ^3^Laboratório de Genética, Instituto Butantan, 05503-900 São Paulo, SP, Brazil; ^4^Laboratório de Virologia, Instituto Butantan, 05503-900 São Paulo, SP, Brazil; ^5^Laboratório de Virologia, Universidade Federal de Goiás, 75801-615 Jatai, GO, Brazil; ^6^Departamento de Parasitologia, Instituto de Ciências Biomédicas, Universidade de São Paulo, 05389-970 São Paulo, SP, Brazil; ^7^São Lucas Medical School, Rua Alexandre Guimarães 1928, 76000-000 Porto Velho, RO, Brazil; ^8^Instituto de Pesquisa Ecológica (IPE), 12960-000 Nazaré Paulista, SP, Brazil; ^9^Divisão Técnica de Medicina Veterinária e Manejo da Fauna Silvestre (DEPAVE-/SVMA), 04030-000 Prefeitura de São Paulo, SP, Brazil; ^10^Departamento Municipal de Saúde, 11950-000 Prefeitura de Cajati, SP, Brazil; ^11^Centro de Pesquisa em Virologia da Faculdade de Medicina de Ribeirão Preto da Universidade de São Paulo, 14049-900 Ribeirão Preto, SP, Brazil

## Abstract

Brazil has reported more than 1,600 cases of hantavirus cardiopulmonary syndrome (HPS) since 1993, with a 39% rate of reported fatalities. Using a recombinant nucleocapsid protein of* Araraquara* virus, we performed ELISA to detect IgG antibodies against hantavirus in human sera. The aim of this study was to analyze hantavirus antibody levels in inhabitants from a tropical area (Amazon region) in Rondônia state and a subtropical (Atlantic Rain Forest) region in São Paulo state, Brazil. A total of 1,310 serum samples were obtained between 2003 and 2008 and tested by IgG-ELISA, and 82 samples (6.2%), of which 62 were from the tropical area (5.8%) and 20 from the subtropical area (8.3%), tested positive. Higher levels of hantavirus antibody were observed in inhabitants of the populous subtropical areas compared with those from the tropical areas in Brazil.

## 1. Introduction

Hantaviruses are emerging pathogens that have gained increasing attention in the last few decades [1]. The genus* Hantavirus* belongs to Bunyaviridae family and is transmitted to human by rodents and possible by other small mammals. More than 40* Hantavirus* species are currently known and 22 of them are considered pathogenic for humans [2]. The hantaviruses found in Eurasia (e.g.,* Hantaan* and* Seoul* virus) are harbored by rodents of the Murinae and Arvicolinae subfamilies and cause hemorrhagic fever with renal syndrome (HFRS) in infected humans. On the other hand the hantaviruses found in the Americas (e.g.,* Sin Nombre*,* Juquitiba*, and* Castelo dos Sonhos*) are harbored by rodents of the Sigmodontinae subfamily and cause Hantavirus Pulmonary Syndrome (HPS) in humans [3–5].

As Manigold and Vial [2] wrote, moles, shrews, and bats are also increasingly described as natural hosts of new members of the* Hantavirus* genus (e.g.,* Huangpi* virus,* Lianghe* virus,* Longquan* virus,* Yakeshi* virus, and* Seewis* virus). However, the pathogenicity of these viruses for humans is unclear. Also, there are reports of seropositive domestic animals such as dogs and cats, suggesting that these become infected from contact with infected primary hosts. Another interesting study fresh published [5], demonstrates pet rats and whales at United Kingdom. However, there is neither evidence of disease in these species nor of a role as a reservoir for human infection.

In Brazil the HPS cases are mostly caused by five genotypes of hantavirus:* Juquitiba* virus (JUQV),* Araraquara* virus (ARAV),* Laguna Negra-like* virus (LANV-like),* Castelo dos Sonhos* virus (CASV), and* Anajatuba* virus (AJBV) [5]. A significantly higher number of HPS-associated fatalities (50%) were observed in the Midwestern and Southwestern regions compared with other regions of Brazil [6, 7]. Serological evidence of HPS has also been reported in the north and northeast of Brazil where hantavirus genotypes are unknown [5–10].

The first confirmed cases of HPS in North America occurred in 1993, and six months later, it was reported in Brazil [5, 11, 12]. Since then, more than 1600 HPS cases have been reported in Brazil by Brazilian Ministry of Health/SVS, with approximately 39% being fatalities [13].

Serological methods are commonly used for hantavirus diagnosis, including enzyme-linked immunosorbent assays (ELISAs), immunofluorescence assays, and immunoblot assays. Additionally, hantavirus isolation in Vero E6 cell cultures and detection of anti-hantavirus antibodies by plaque reduction neutralization are also used for diagnosis. Nevertheless, both methods require labor-intensive, time-consuming, and biosafety-level-three conditions [6]. On the other hand, molecular biology such as RT-PCR allows molecular characterization (viral genotype) and accurate diagnosis [14].

Here, in an effort to better understand and study the distribution of infections by hantavirus in Brazil, we present the results of a serological survey including individuals living in urban and rural areas near the Amazonian forest and in subtropical areas near rain forests that have degraded environmental conditions. Sera from the participants were tested via an IgG-ELISA [15] that uses a recombinant nucleocapsid protein from ARAV as the antigen [16].

## 2. Material and Methods

### 2.1. Sites and Study Population

**Figure 1:**
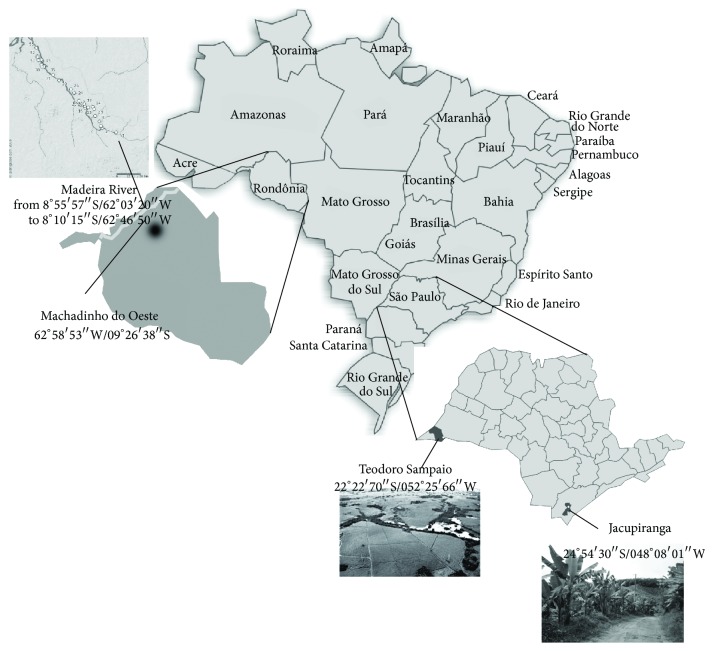
Map of Brazil showing the four study sites: the city of Jacupiranga (RV) and Teodoro Sampaio (PP) in São Paulo state in the subtropical region and Machadinho do Oeste city and the Machado river region Rondônia state in the tropical region.

The design for minimum sample size was performed in accordance with the calculations specified by Luiz and Magnanini [17]. Based on the presence of wild rodents cohabiting with humans and the occurrence of HPS cases, four study sites were selected for this serological survey between 2003 and 2008. Machado river (from 8°55′57′′S/62°03′20′′W to 8°10′15′′S/62°46′50′′W) and Machadinho do Oeste county (09°26′38′′S/61°58′53′′W) are both in Rondônia state in the Amazon tropical region. In 2003, 435 participants living along the Machado river and working on subsistence farming were enrolled and subjected to blood drawing for the study (Figure 1). In Machadinho do Oeste county, 633 inhabitants were enrolled and subjected to blood drawing in 2005. These participants lived in an urban area surrounded by tropical Amazonian forests (Figure 1). The other two study sites were located more than 2000 km away, near the subtropical rain forests of Sao Paulo state. These regions included Jacupiranga county in the Ribeira Valley, where 65% of the Brazilian Atlantic forest remains (24°54′30′′S/048°08′01′′W). A total of 157 inhabitants working on banana or orange farms and cattle or fish raising were enrolled in Jacupiranga in 2007 and subjected to blood draws (Figure 1). The fourth site was in Teodoro Sampaio county (22°22′70′′S/052°25′66′′W) at the mouth of the Paranapanema river, where the land has been subject to disorganized occupation and massive deforestation. Currently, farms at the mouth of the Paranapanema river have intensive agricultural activity (Figure 1). In Teodoro Sampaio, 85 inhabitants were enrolled in the study in 2008 and subjected to blood draws. All clinical samples were transported to the laboratory in nitrogen liquid and stored at −80°C.

### 2.2. Ethical Considerations

The enrollment of participants in this serological survey was authorized by the Ethics Committee of ICB/USP (670/2005), and the confidentiality of their personal information was ensured. Blood collection of the participants was only performed after signing the informed consent, in compliance with the rules of the ethics committee. After participants signed the consent form, the survey was applied for evaluation of the risk factors, gender, age, education, and another aspects as epidemiological information.

### 2.3. ELISA

Sera were tested by an indirect IgG enzyme-linked immunosorbent assay (ELISA) using the N recombinant protein (recN) of ARAV as antigen, as described by Figueiredo et al. [15, 16], with some modifications. Briefly, 96-well microplates (PolySorp*™*, Nunc, USA) were coated with 50 *μ*L of ARAV recN protein or the respective control (0.5 *μ*g/mL) overnight in a wet chamber at 4°C. Both antigens were diluted in carbonate-bicarbonate buffer (0.05 M, pH 9.6). All incubations were conducted at 37°C for 1 h, and the plates were washed six times with wash buffer (phosphate-buffered saline [PBS]-0.1% Tween 20) between each step. In the first step, 50 *μ*L of a blocking solution containing 5% bovine serum albumin in PBS (Sigma, San Francisco, CA, USA) was added to each well, and the plate was incubated for 2 h in a moist chamber at 37°C. Next, 50 *μ*L of the serum sample diluted at 1 : 100 or control samples diluted to 1 : 1000 in dilution buffer (PBS/BSA 1%) were added per well. In the third step, the wells were incubated with 50 *μ*L of phosphatase-labeled anti-human IgG antibody (Sigma, San Francisco, USA). Finally, 1 mg/mL of nitrophenyl phosphatase substrate (pNPP, Sigma, USA) was added per well, and the reaction was stopped after 20 minutes by adding 50 *μ*L of 3 M NaOH. The absorbance was measured at 405 nm using a Multiskan MS (Labsystems, Helsinki, Finland). The cut-off was established as the mean value + 2 standard deviations control samples and showed a cut-off Optical Density (OD) equal to or greater than 0.500.

The recN protein used as the antigen and the positive/negative control samples were kindly provided by the Laboratories of Arboviruses and Rodent-borne Viruses of the University of São Paulo School of Medicine (Luis Tadeu M. Figueiredo, Ph.D., M.D.).

### 2.4. Data Analysis

A statistical analysis of the results was performed with Prism version 5.0 (GraphPad Software). Associations of positive serological tests with subtropical areas and demographic and socioeconomic variables were analyzed using the Chi-square test, and *p* < 0.05 was considered significant.

## 3. Results and Discussion

**Figure 2:**
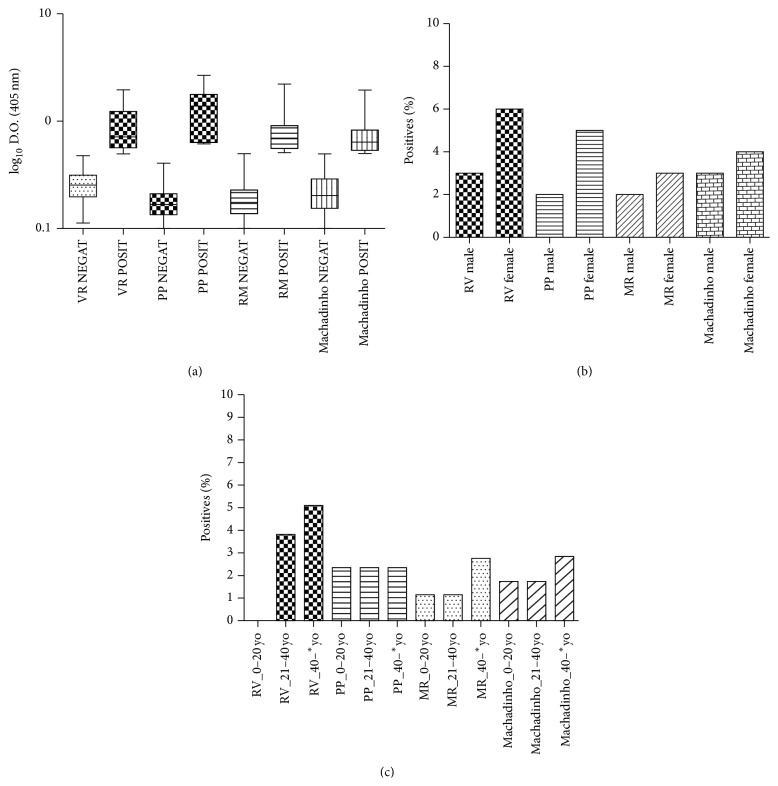
(a) demonstrated the absorbance values by ELISA for the total positive (POSIT) and negative (NEGAT) individuals from four different sites (Jacupiranga, SP, in Ribeira Valley, VR; Teodoro Sampaio/SP in Pontal Parapanema, PP; Machado River/RO, RM; and Machadinho do Oeste/RO) for the presence of anti-hantavirus IgG antibodies. (b) The graphic represent results from this four study sites in the tropical and subtropical region (Jacupiranga/SP in Ribeira Valley, VR; Teodoro Sampaio/SP in Pontal Parapanema, PP; Machado River/RO, RM; and Machadinho do Oeste/RO), categorized by gender. (c) The graphics represent results from this four study sites in the tropical and subtropical region (Jacupiranga/SP in Ribeira Valley, VR; Teodoro Sampaio/SP in Pontal Parapanema, PP; Machado River/RO, RM; and Machadinho do Oeste/RO) categorized by age (groups of 20 years). *∗* means “or more.”

Sera from all 1,310 participants in this study were tested using the ARAV recN-ELISA, and 82 (6.2%) showed positive results. In the Machado river group (MR), 22 (1.6%) of the 435 participants examined had IgG antibodies to hantavirus. In the Machadinho do Oeste county, 40 (3.0%) among the 633 participants analyzed were previously infected by hantavirus. In Jacupiranga county (RV) in the southeastern part of Brazil, 14 (9.0%) of the 157 participants had IgG antibodies to hantavirus. In Teodoro Sampaio county (PP) in the São Paulo state, 6 (7.0%) of the 85 participants were seropositive for hantavirus, as shown in Figure 2(a).

Participants from Jacupiranga and Teodoro Sampaio counties in the subtropical southeastern region of Brazil were significantly (*p* = 0.0008) more seropositive to hantavirus (20/242, 8.3%) than those from the tropical Amazonian regions of Machado river and Machadinho do Oeste county (62/1068, 5.8%).

Among the hantavirus seropositive, 60.9% were female participants, as shown in Figure 2(b), consisting of 877 women and 433 men (50 and 32, resp., were seropositive). In Machadinho do Oeste county, it was observed that 3.63% of females and 2.68% of males were seropositive. In MR it was observed that 3.22% of females and 1.84% of males were seropositive. In RV, it was observed that 5.73% of females and 3.18% of males were seropositive.

The ages of those seropositive to hantavirus ranged from five to 82 years but included mostly young adults greater than 20 years of age, as shown in Figure 2(c).

The seropositivity to hantavirus was not correlated with education level, place of birth, time of residence in the study site, or other risk factors evaluated for epidemiological analyses.

In this study, we evaluated the hantavirus seroprevalence in people living in areas presumed of high risk for this infection. A high proportion of hantavirus seropositive subjects were observed in participants from Jacupiranga county (9%) and Teodoro Sampaio (7%), both of which located at subtropical regions of the state of São Paulo. This value is significantly higher than the 1.6% of seropositive cases reported in a serological survey in the Ribeira Valley performed in 1993 [8, 12].

The seroprevalence of 6% and 8% to hantavirus observed in the present study is not outside standards range observed in the literature, like, for example, the rate of 14% observed in the population of Jardinopolis county in the state of Sao Paulo [18] and of 6.1% in Chile, presumably, with ANDV [19]. Higher seroprevalence was observed, presumably, to the genotype ORNV, in northern Argentina (20%), and, presumably, to the genotype LNV in Paraguayan Chaco, where 12.8% of the urban population and 57% of the indigenous population were infected [20].

In the tropical Amazonian region of Rondônia, the seroprevalence to hantavirus in Machadinho do Oeste county (3%) and Machado river region (2.8%) was similar. Other studies conducted in the Amazon region have yielded different hantavirus seroprevalence rates. A survey of several cities in the Amazonas state between 2007 and 2009 yielded a seroprevalence rate of 0.6% [11], whereas another study performed along a highway that crosses the Amazon region from Cuiabá city to Santarem city reported seroprevalence rates of 2.16% to 9.43% [21].

According description by Nava et al. [22], in the Pontal do Paranapanema, at Morro do Diablo State Park (Teodoro Sampaio, SP), it is a forested region, surrounded by small rural settlements and large private properties. The area is a wedge-shaped region bounded on the north by Rio Paraná and on the south by Rio Paranapanema. As well as the Ribeira Valley (Jacupiranga, SP), the Atlantic Rain Forest in São Paulo state is one of the last remaining significant zones, where only 1.8% of the original natural vegetation remains and is considered a biodiversity hotspot.

A significantly higher proportion of seropositive individuals to hantavirus was observed in the subtropical region of Brazil (São Paulo state) compared to the tropical region (Rondônia state). It is possible that the higher occurrence of hantavirus infections in São Paulo state is associated with the degradation of the local environment. São Paulo, which is the most densely populated state in Brazil, has two main ecosystems: the “cerrado (savannah)” in its western region and neotropical Atlantic Rain Forests along the coast. These ecosystems sustain Sigmodontinae rodents and have been modified, segmented, and damaged by extensive sugarcane, soybean and coffee farming, livestock raising, and rapid and poorly planned urbanization. Such degraded landscapes allow close contact of humans with zoonoses, resulting in enhanced transmission of pathogens to humans. Environmental degradation favors the abundance of opportunistic rodent species (*Necromys lasiurus, Akodon* sp., and* Calomys tener*), which are risk factors for hantavirus infection. In contrast, the environment and landscape in the Amazonian tropical region (Machado river region and Machadinho do Oeste county) is better conserved and has a higher diversity of rodents. Therefore, the participants from these study sites were less frequently infected by hantavirus given that the biodiversity loss would tend to increase pathogen transmission.

One of the limitations of this study is the seroprevalence accuracy of data available at these areas combined with full background from participants of this study. Also, it was impossible to analyze hantavirus infection in rodents from the same studied sites since we did not collect samples. Probably, a comparison of seropositivity to hantavirus among humans and rodents at the different study sites would help to understand why more infections occur in the subtropical area of Brazil (São Paulo state) more than in the tropical area (Rondônia state).

## 4. Conclusions

In conclusion, our findings highlight a higher seroprevalence rate (IgG) for antibodies against hantavirus in the human population in Brazil, with a higher rate in the subtropical region (Atlantic Rain Forest) than in the tropical region (Amazon Forest). Degraded ecosystems allow close contact of humans with zoonoses, resulting in enhanced transmission of pathogens to humans. Particularly it is relevant because São Paulo state is one of the most densely populated states in Brazil. We are highlighting our findings to provide a better understanding of hantavirus infection and circulation in Brazil, specifically demonstrating hotspots that will require public health action to prevent a possible outbreak.

## References

[1] Zeier M., Handermann M., Bahr U. (2005). New ecological aspects of hantavirus infection: a change of a paradigm and a challenge of prevention—a review. *Virus Genes*.

[2] Manigold T., Vial P. (2014). Human hantavirus infections: epidemiology, clinical features, pathogenesis and immunology. *Swiss Medical Weekly*.

[3] Schmaljohn C. S., Nichol S. T., Knipe D. M., Howley P. M. (1997). Bunyaviridae. *Fields Virology*.

[4] Nichol S. T., Arikawa J., Kawaoka Y. (2000). Emerging viral diseases. *Proceedings of the National Academy of Sciences of the United States of America*.

[5] Jameson L. J., Taori S. K., Atkinson B. (2013). Pet rats as a source of hantavirus in England and Wales, 2013. *Eurosurveillance*.

[6] Jonsson C. B., Figueiredo L. T. M., Vapalahti O. (2010). A global perspective on hantavirus ecology, epidemiology, and disease. *Clinical Microbiology Reviews*.

[7] Figueiredo L. T. M., Moreli M. L., Campos G. M., Sousa R. L. M. (2003). Hantaviruses in São Paulo State, Brazil. *Emerging Infectious Diseases*.

[8] Figueiredo L. T. M., Moreli M. L., De Sousa R. L. M. (2009). Hantavirus pulmonary syndrome, central plateau, southeastern, and southern Brazil. *Emerging Infectious Diseases*.

[9] Suzuki A., Bisordi I., Levis S. (2004). Identifying rodent hantavirus reservoirs, Brazil. *Emerging Infectious Diseases*.

[10] Mendes W. S., da Silva A. A. M., Aragão L. F. C. (2004). Hantavirus infection in Anajatuba, Maranhão, Brazil. *Emerging Infectious Diseases*.

[11] Gimaque J. B. L., Bastos M. S., Braga W. S. M. (2012). Serological evidence of hantavirus infection in rural and urban regions in the state of Amazonas, Brazil. *Memorias do Instituto Oswaldo Cruz*.

[12] Plyusnin A., Morzunov S. P. (2001). Virus evolution and genetic diversity of hantaviruses and their rodent hosts. *Hantaviruses*.

[13] Iversson L. B., da Rosa A. P., Rosa M. D., Lomar A. V., Sasaki M. G., LeDuc J. W. (1994). Human infection by Hantavirus in southern and southeastern Brazil. *Revista da Associacao Medica Brasileira*.

[14] Guterres A., de Oliveira R. C., Fernandes J., Schrago C. G., de Lemos E. R. S. (2015). Detection of different South American hantaviruses. *Virus Research*.

[15] Figueiredo L. T. M., Moreli M. L., Borges A. A. (2009). Evaluation of an enzyme-linked immunosorbent assay based on Araraquara virus recombinant nucleocapsid protein. *American Journal of Tropical Medicine and Hygiene*.

[16] Figueiredo L. T. M., Moreli M. L., Borges A. A., Figueiredo G. G., Souza R. L. M., Aquino V. H. (2008). Expression of a hantavirus N protein and its efficacy as antigen in immune assays. *Brazilian Journal of Medical and Biological Research*.

[17] Luiz R. R., Magnanini M. M. (2000). A lógica da determinaçäo do tamanho da amostra em investigaçöes epidemiológicas. *Cadernos de Saúde Coletiva*.

[18] Campos G. M., De Sousa R. L. M., Badra S. J., Pane C., Gomes U. A., Figueiredol L. T. M. (2003). Serological survey of hantavirus in Jardinopolis County, Brazil. *Journal of Medical Virology*.

[19] Frey M. T., Vial P. C., Castillo C. H., Godoy P. M., Hjelle B., Ferrés M. G. (2003). Hantavirus prevalence in the IX Region of Chile. *Emerging Infectious Diseases*.

[20] Ferrer J. F., Jonsson C. B., Esteban E. (1998). High prevalence of hantavirus infection in Indian communities of the Paraguayan and Argentinean Gran Chaco. *American Journal of Tropical Medicine and Hygiene*.

[21] Medeiros D. B. A., da Rosa E. S. T., Marques A. A. R. (2010). Circulation of hantaviruses in the influence area of the Cuiabá-Santarém Highway. *Memorias do Instituto Oswaldo Cruz*.

[22] Nava A. F. D., Cullen L., Sana D. A. (2008). First evidence of canine distemper in brazilian free-ranging felids. *EcoHealth*.

